# “Insight into who you are as a human being”: Perceptions of the Utility and Usability of a Values Assessment Tool (VAsT) for Women With Metastatic Breast Cancer

**DOI:** 10.1177/10732748261466394

**Published:** 2026-07-28

**Authors:** Robert Crowder, Victoria Crowder, Soroush Fariman, Natasha Joglekar, Danielle Anderson, Juanita Miller, Stephanie Walker, Emily M. Ray, Shaunta Ford-Pierce, Bryce B. Reeve, Antonia Bennett, Melissa Mazor, Allison M. Deal, Lorinda A. Coombs

**Affiliations:** 1 2331 Office of Human Research and Ethics, University of North Carolina at Chapel Hill, Chapel Hill, NC, USA; 2 School of Nursing, University of North Carolina at Chapel Hill, Chapel Hill, NC, USA; 3 Division of Pharmaceutical Outcomes and Policy, University of North Carolina at Chapel Hill, Chapel Hill, NC, USA; 4 6558 Lewis Katz School of Medicine, Temple University, Philadelphia, PA, USA; 5 University of North Carolina at Chapel Hill, Chapel Hill, NC, USA; 6 School of Medicine, Division of Oncology, North Carolina Basnight Cancer Hospital, Lineberger Comprehensive Cancer Center, Chapel Hill, NC, USA; 7 3065 School of Medicine, Duke University, Durham, NC, USA; 8 Division of General Internal Medicine, Icahn School of Medicine at Mount Sinai, New York, NY, USA

**Keywords:** metastatic breast cancer, values assessment, communication, community advisory board, caregivers, qualitative, shared decision making, patient-centered communication, older adults, values clarification, patient preferences, humanistic communication

## Abstract

**Introduction:**

Women with metastatic breast cancer (mBC) often face complex treatment decisions that require careful consideration of values and priorities, which may be unique to each individual and evolve over the course of treatment. The patient-reported Values Assessment Tool (VAsT) was developed to help prioritize and communicate values to clinicians and care partners, thereby improving treatment decision-making. This study aimed to further refine the VAsT, assess perspectives on utility and usability of the VAsT, and evaluate the potential impact on communication between women with mBC, care partners, and clinicians who utilize the VAsT.

**Methods:**

A qualitative study using cognitive interview methodology was conducted. Qualitative cognitive interviews were conducted with seven women with mBC, three care partners, and four breast oncology clinicians. Participant feedback informed the refinement of the tool, and thematic analysis guided by Normalization Process Theory was conducted to understand the utility and usability of the VAsT among women with their clinicians and care partners.

**Results:**

The VAsT domains were confirmed (e.g., *financial concerns of affording cancer care; minimize and manage side effects of treatment*), and instructions and purpose were refined. Three themes related to the usability and communication with VAsT were identified: 1) Comprehension of values and meaningful use of the tool, 2) Usability and format of the VAsT, and 3) Communication and clinical utility of the VAsT.

**Conclusion:**

Findings indicate there is variation in perspectives of how often the VAsT should be utilized over the course of treatment, which future research on the feasibility and efficacy of the VAsT will better elucidate. Women with mBC may use the VAsT to facilitate communication and build trust with clinicians, which is perceived to allow for higher-quality care and value-congruent treatment decisions.

## Introduction

It is estimated that over 150,000 women in the United States live with metastatic breast cancer (mBC) each year,^1^ and this number is increasing due to advances in treatment.^2^ While mBC remains incurable, the past decades have seen significant increases in 5-year survival rates and decreases in mortality.^2^ For women with mBC, care plans must account for periods of no evidence of active disease and disease progression, treatment tolerability, and social-emotional considerations, including the inclusion of care partners and the patient’s preferences and values over time.^3^ While that is not always possible given treatment-related toxicity or other individualized circumstances, patient values should be strongly considered in plans of care.

Integrating patients’ values into their treatment decisions and care plans is an important yet overlooked aspect of cancer care, which may be addressed by improving communication with providers. When navigating options, women with mBC may make decisions that are strongly driven by their values (e.g., increased survival or maintaining quality of life), even if this comes with other drawbacks or an increased symptom burden.^4^ It is particularly important to consider the perspectives of women with mBC, distinct from those of women with non-metastatic cancer, because values in care differ.^3,5,6^ However, prior research has shown that clinicians may focus on the next steps in treatment and not initiate conversations about what the person values,^7,8^ which requires patients to advocate for themselves in care discussions. Effective communication among women with mBC, their clinicians, and care partners about what women with mBC value in their treatment is important to the shared-decision-making process. Improving communication is a critical function of shared-decision-making tools,^9,10^ and can improve clinical outcomes.^11,12^

As detailed in a previously published paper,^13^ researchers developed domains for a Values Assessment Tool (VAsT) to enhance communication of values among women with mBC regarding treatment decisions with their care partners and clinicians. Nine domains were identified that captured the values of women with mBC in their treatment. For example, women noted that valuing their *quality of life* has impacted their treatment decisions or *living to care for a loved one*. After the initial VAsT development, the tool needed further refinement. This included refining the tool’s domains, usability, and exploring the utility of the VAsT for women with mBC, their care partners, and clinicians in guiding their treatment decisions. This study distinguishes between *utility*, defined as the functionality of the VAsT (e.g., assessment timing, potential to impact communication), and *usability*, defined as how easy it is for participants to use the VAsT.

### Study Purpose and Aims

The purpose of this study was to further refine the VAsT and assess perspectives on the utility and usability in clinical practice. The aims were, from the perspectives of women with mBC, care partners, and breast care clinicians: 1) Confirm the domains, or change them if necessary, to refine the tool; 2) Explore VAsT’s utility and usability in an outpatient oncology setting; and 3) Explore the potential impact of VAsT on communication between women, care partners, and clinicians.

## Methods

A qualitative study using cognitive interview methodology was conducted to explore VAsT’s utility in the oncology outpatient clinical setting, its potential usability in communicating with clinicians, and how to further refine the tool. This study builds on prior formative work to develop the VAsT.^13^ Data collection, analysis, and tool refinement were conducted with input from a community advisory board (CAB) comprised of two women advocates with mBC, one care partner, and two breast oncology clinicians. The University of North Carolina at Chapel Hill (UNC-CH) Institutional Review Board (IRB) and the Lineberger Comprehensive Cancer Center Protocol Review Committee reviewed and approved this study in 2022 (IRB 22–1806), which was conducted in accordance with the Helsinki Declaration of 1975, as revised in 2024. The reporting of this study conforms to the COREQ guidelines.^14^

### Participants

Women with mBC and care partners were purposively recruited with assistance from CAB members. Eligibility criteria for women with mBC were: (1) diagnosis of mBC; (2) age ≥18 years; (3) ability to understand and speak English; and (4) ability to participate in study procedures. Women unable to provide consent or receiving hospice care were excluded. Care partners were identified by the person diagnosed with mBC. All participants provided written informed consent before participating in study activities.

In-person recruitment was conducted by study staff in outpatient clinics at UNC-CH, a comprehensive cancer center in the Southeastern U.S. Additional recruitment was conducted through outreach to Mount Sinai Medical Center, a comprehensive cancer center in the Northeastern U.S., and by CAB members sharing recruitment materials with support groups for women with mBC. Clinicians who actively provide care to women with mBC at UNC-CH were recruited via email invitation from the principal investigator [LC]. Clinicians were purposefully selected for their expertise in working with women with mBC and for their diverse roles on the care team (e.g., nurse navigator, nurse practitioner, physician, and physician assistant).

### Values Assessment Tool (VAsT)

The Values Assessment Tool (VAsT) identified nine unique domains which are significant values for women with metastatic breast cancer when making treatment decisions: *1) desire to not appear sick; 2) desire to support others by participating in clinical research; 3) financial concerns to afford cancer care; 4) living to care for a loved one; 5) maintain sexuality and minimize impact on intimate relationships; 6) maintain quality of life during treatment; 7) maximize time away from medical appointments; 8) minimize and manage side effects of treatment;* and *9) stop or slow disease progression with an effective treatment plan including the patient.*^13^ Based on formative qualitative interviews, prior tool-development research,^15,16^ and input from a CAB, the VAsT was developed as a rank-order tool.

The VAsT instructions were for women with mBC to rank the nine domains of value in mBC treatment. The instructions read: “*Please place the categories below from most (1) to least (9) important for you to make decisions about your cancer treatment. This tool can be used at any point during your treatments. We recommend to re-assess at any point as you want or need.*”

### Data Collection

Cognitive interviews lasting approximately 30 minutes were conducted via video teleconferencing by two members of the research team from December 2024 to April 2025 [LC, VC]. The interviews followed prior methods of survey development, which include cognitive interviews to refine the items of a tool, such as the VAsT.^15,17,18^ The purpose of interviews with women with mBC and care partners was to ensure clarity of the domains and the tool, to understand its usability, and its utilization. With these participants, interview questions used a “think-aloud” method in which participants self-administered the VAsT and verbalized their thoughts on clarity and recommended revisions, with semi-structured follow-up questions from the interviewer (Supplementary File).^18^ Interviews with clinicians focused on which items would be most useful for informing their discussions with patients and how to integrate the VAsT with their usual clinical workflows. Interview guides were pilot tested and reviewed with the research team after the first interview for each participant type (women with mBC, care partners, and clinicians). Interviews were audio-recorded, transcribed verbatim using a professional transcription service, de-identified, and uploaded to Dedoose software (Version 10.0.59, Los Angeles, CA)^19^ for analysis. Patients and care partners received a $75 gift card for their time.

Researchers who conducted the interviews [LC, VC] were both female clinicians with clinical, academic, and research backgrounds. Both were employed as nurse researchers, and LC additionally as a nurse practitioner. LC had extensive clinical experience with women diagnosed with breast cancer. Both researchers had experience conducting qualitative interviews in clinical settings. Before participating in the study, participants did not have established relationships with the researchers. The researchers provided minimal personal details and instead focused the conversation on the study’s purpose and overall goals of the VAsT during the informed consent process.

### Statistical Analysis

Initial data analysis was conducted to revise the VAsT for use in a future pilot study, using methods aligned with prior cognitive interview analyses.^16-18^ Two researchers [LC, VC] reviewed transcripts and independently identified (a) positive items and trends, (b) problem items and trends, and (c) problems identified by a few participants but which may affect VAsT outcomes. Discrepancies were discussed, the transcripts were reviewed, and changes to the VAsT were organized in a word processing item-tracking sheet. The proposed changes were presented to the CAB members for input and discussed with the CAB and the research team.

The second phase of data analysis was thematic analysis, conducted by five team members [RC, VC, SF, NJ, DA] using Dedoose software to apply codes.^19,20^ The purpose of thematic analysis was to understand the usability and utility of the VAsT and its impact on communication for women with their clinicians and care partners. Transcripts were coded independently by two researchers, and discrepancies were resolved through discussion and, if needed, by an independent researcher [LC]. Memos were used to document the analysis process as an audit trail. Because of the goal for VAsT to be implemented in clinical practice, Normalization Process Theory (NPT)^21^ was used to guide analysis. NPT is a theory used to guide the implementation of interventions in clinical practice, with four main constructs: *coherence* (making sense of the intervention); *cognitive participation* (engaging with the intervention); *collective action* (working to conduct the intervention); and *reflexive monitoring* (assessing and evaluating the intervention.^21^ For example, in this study, the concept of *coherence* was included as a code to understand the meaning of the tool and its ease of use. Additionally, NPT concepts were linked to each theme generated. The research team developed a thorough understanding of the perspectives of women with mBC, care partners, and breast care clinicians on the VAsT and reached meaning saturation with 14 participants.^22^ Themes were identified by the researchers and refined with input from the CAB members.

## Results

Cognitive interviews were conducted with seven women with mBC, three care partners, and four breast oncology clinicians. Demographic characteristics are described in Table 1. One woman with mBC declined for unknown reasons, with multiple attempts to reach her. All enrolled women with mBC identified as female, and the average age was 58 years (range 33-76). Most women had been diagnosed with mBC from 1-3 years (n=4, 57%) and were white (n=4, 57%). All enrolled care partners identified as male, and 2/3 were married. Clinician demographic data are not reported to avoid identifying individual clinicians from a small population of breast oncology clinicians at the participating site. Clinicians represented disciplines including the roles of nurse navigator, nurse practitioner, physician, and physician assistant.

**Table 1.** Demographic Characteristics of Women With mBC (n=7) and Care Partners (n=3)

Characteristic	N (%)	N (%)
	Women with mBC	Care partners
Age (Mean, range)	58 (33-76)	56 (range not reported)
Age (years)		
30-39	1 (14%)	-
50-59	3 (43%)	-
60-69	1 (14%)	-
>70	2 (29%)	-
Diagnosis Length, years		
1-3	4 (57%)	-
>7	2 (29%)	-
Origin of participants		
Southeastern US	4 (57%)	2 (67%)
Northeastern US	3 (43%)	1 (33%)
Sex		
Female	7 (100%)	0
Male	0	3 (100%)
Race		
White	4 (57%)	2 (67%)
Black or African American	3 (43%)	1 (33%)
Ethnicity		
Not Hispanic or Latino	6 (86%)	2 (67%)
Hispanic or Latino	1 (14%)	1 (33%)
Sexual orientation		
Straight/Heterosexual	7 (100%)	3 (100%)
Marital status		
Married/Partnered	2 (29%)	2 (67%)
Single	2 (29%)	1 (33%)
Divorced	2 (29%)	0
Domestic partnership	1 (14%)	0
Employment status		
Full-time	0	3 (100%)
Unemployed	1 (14%)	0
Retired	3 (43%)	0
Other	3 (43%)	0
Highest level of education		
High school graduate, GED	2 (29%)	0
Some college, no degree	0	1 (33%)
College and/or advanced degree	5 (71%)	2 (67%)
Annual household income		
$12,900-24,999	4 (57%)	0
$50,000-$74,999	2 (29%)	1 (33%)
>$75,000	1 (14%)	2 (67%)

Three themes were identified: 1) Comprehension of values and meaningful use of the tool; 2) Usability and format of the VAsT; and 3) Communication and clinical utility of the VAsT. Details on themes, subthemes, exemplar quotations, and connections to the NPT concepts are provided in Table 2. Revisions made to the VAsT based on study findings are detailed below, and in Table 3.

**Table 2.** Themes and Exemplar Quotations Regarding Values Assessment Tool (VAsT) Utility and Relevance Regarding Treatment Decisions for Women With Metastatic Breast Cancer

Normalization Process Theory (NPT) concept	Theme	Sub-theme	Exemplar quotations
*Coherence; and Cognitive participation*	Comprehension of values and meaningful use of the tool	Using personal experiences to understand values and their changes with the context of metastatic disease	“I would say I think it really depends on the health of the patient and their desires, and what they want to do with their time in terms of maintaining sexuality, of course, and minimizing impact on relationships.” [Care partner, ID70]
			“Four would be the financial concerns because I think it, about that time, that’s goin’ to start really hitting you that how are we gonna do this? How could we do this and still live and eat and pay bills and—because in today’s world, you’ve got husband and wife or partners are both working. You got one income going away. That’s gonna be a question.” [Care partner, ID73]
		Understanding clarity of the instructions and purpose of the VAsT	“This [VAsT] would be a different form by comparison to all that other stuff that’s in that bucket.” [Woman with mBC, ID15]
			“Yeah, what am I supposed to do, answer it like I have been or …?” [Woman with mBC, ID19]
			“I think the directions are clear… That’s pretty straightforward. Yeah, I don’t have any qualms with how this is written and the instructions provided.” [Care partner, ID70]
*Collective action*	Usability and format of the VAsT	Electronic use of the tool by women with mBC and clinicians	“I think it’ll be nice for it to be electronic, of course. I think that would be the simplest way, but then we have to consider our patients who don’t use the electronic record. I think havin’ the option of havin’ the tool as a paper at the time of the visit for the patient to complete.” [Clinician]
			“I definitely think portal on your phone, an app on your phone. Definitely not verbal or some kind of—I just refuse at my doctor’s office a verbal basic questionnaire ‘cause I’m just mentally tired, so I think a paper sometimes can be tiring too. I think the portal would be best.” [Woman with mBC, ID22]
			“What are you gonna do for people that don’t have computers or no electronic device?” [Woman with mBC, ID16]
		Use of the VAsT at the beginning and periodically throughout treatment	“I would do it each and every time. It would be important. I would do it each and every time when I do my check-in.” [Woman with mBC, ID14]
			“I would say somewhere between every three to six months…. Because things for us can change drastically in three to six months.” [Woman with mBC, ID20]
			“I think that it’ll be very good initially, and then maybe if you switch doctors” [Care partner, ID72]
*Reflexive monitoring*	Communication and clinical utility of the VAsT	Potential to enhance bidirectional communication regarding patients’ values in treatment decisions for women with mBC and their clinicians and care partners	[Regarding using the VAsT for communicating with clinicians] “When I first was diagnosed I never got involved in anything. I just went along with the program. Today is a different day and I’m a different woman.” [Woman with mBC, ID14]
			“Well, I, sometimes—even though I have a very open communication with my doctors, sometimes we can skip some stuff, or we don’t talk about—probably he doesn’t know about my financial concerns.” [Woman with mBC, ID24]
			“I think we have these conversations, but this could even help guide it before we have the talk. I think we talk about hair loss something that for some people’s important, for others less important. Time away from appointments when we’re thinking about different treatments. They have different schedules.” [Clinician]
		Perceived clinical relevance of the VAsT	“It’s like I go there for one single purpose. Tell me what’s going on with my PET scan…I would tell her about this [VAsT], but it’s not something that when I go to see her, that we talk. We don’t talk about this stuff.” [Woman with mBC, ID14]
			“Well, it’s [VAsT] making your doctors and your clinicians aware of your concern, so they’ll talk to you about them and help—maybe help resolve some of the issues.” [Woman with mBC, ID16]
			“I think that they—if they had this information, it would save time, instead of prying into someone and asking a thousand questions. The doctor can say, “The first three, let’s—“He’ll in his mind—already know, one, two, three, let’s help this lady with these concerns.” [Woman with mBC, ID16]
			“I think these questions are worded that way to where it gives the patient/caregiver a tool to come back and say, “Look, this—I’ve got these financial concerns. How are we gonna—how can you help me with this? Are there options?”” [Care partner, ID73]
		Perceived quality of care, relational context, and trust in the care team as determinants of VAsT utility	“As you’re getting to know your doctor, it would be nice if you filled this out, and he was made aware of all these before he met you, because it helps you get to know a person.” [Woman with mBC, ID16]
			“If you haven’t already built some type of relationship with your provider, I think this tool gives your provider an insight into who you are as a human being, rather than a patient and just a case. I think it just provides them with a bit more of a human side to you, so that maybe they can also when it comes to making choices and treatment decisions for you, that they are able to see a little bit of that.” [Woman with mBC, ID20]
			“It was missed for two and a half years…Then because they never read the pathology report and I was having mammograms and ultrasounds all along, and it still was missed.” [Woman with mBC, ID19]

**Table 3.** Revisions to the Values Assessment Tool (VAsT) Based on Cognitive Interview Findings

Component	Original VAsT*	Changes recommended based on cognitive interview findings	Revised VAsT*
Instructions and purpose	**Instructions:** Please place the categories below from most (1) to least (9) important for you to make decisions about your cancer treatment. This tool can be used at any point during your treatments. We recommend to re-assess at any point as you want or need.	Yes	**Purpose:** The tool's purpose is to identify what the most important considerations are for you in deciding on treatment for metastatic breast cancer.
			**Instructions:** The next page has categories that women with metastatic breast cancer identified as important. Write in the categories from most important (#1) to least important (#9). If you have things that are important to share with your clinician not in the tool, write them in the box at the bottom.
Domain 1	Desire to not appear sick	No	Desire to not appear sick
Domain 2	Desire to support others by participating in clinical research	No	Desire to support others by participating in clinical research
Domain 3	Financial concerns to afford cancer care	No	Financial concerns to afford cancer care
Domain 4	Living to care for a loved one	No	Living to care for a loved one
Domain 5	Maintain sexuality and minimize impact on intimate relationships	No	Maintain sexuality and minimize impact on intimate relationships
Domain 6	Maintain quality of life during treatment	No	Maintain quality of life during treatment
Domain 7	Maximize time away from medical appointments	No	Maximize time away from medical appointments
Domain 8	Minimize and manage side effects of treatment	No	Minimize and manage side effects of treatment
Domain 9	Stop or slow disease progression with an effective treatment plan including the patient	No	Stop or slow disease progression with an effective treatment plan including the patient
Additional domains	None	Yes	**Domain considered but not added:** ‘Support for mental and emotional health’.

*Wording of the domains and instructions has small alterations for copyright; the meaning is unchanged.

### Comprehension of Values and Meaningful Use of the Tool

While participants were prompted to read aloud and complete the VAsT, most women and care partners also shared personal experiences in each domain. In these discussions, participants were developing an understanding of each domain and how it affected treatment decisions.

Many participants discussed upholding their core values, such as advocating for changes to their treatment schedules, within the domain of *Maximizing time away from medical appointments.* Conversely, many acknowledged that values and domain rankings would change over time with the progression of metastatic disease due to shifts in their health and personal circumstances (e.g., aging, financial status, preferences). When ranking the domains, one patient noted:

*“There is a moment—a moment I’m going now, I don’t—I told my doctor, I don’t wanna see anybody else. Don’t send me to a specialist. I just wanna see you. I wanna see my hematologist and I wanna see my gynecologist and that’s pretty much it…All I do is going out, and doctor’s appointments.”* [Woman with mBC, ID24]

Several participants discussed the importance of mental and emotional health support during treatment. As a result of this finding, the research team and CAB considered adding ‘Support for mental and emotional health’ as a domain to the VAsT, but ultimately did not include it due to its perceived poor fit for a treatment decision support tool. Instead, the premise of supporting mental and emotional health is encouraged to be inherent in every treatment decision, rather than a driving factor for selecting one treatment over another.

When asked how to use the VAsT, participants had mixed opinions about the clarity of the instructions and the tool's purpose. Most participants found the instructions clear for ranking the domains, but some were confused or did not see them. Regarding when and how to use the tool, one participant questioned the purpose of the tool: “*I would like to know the purpose of it. Okay, I've ranked these, and then what?*” [Woman with mBC, ID16]. As a result of this finding, the VAsT instructions were separated into ‘*Purpose*’ and ‘*Instructions*’ and moved to a standalone page before participants proceeded with the tool’s ranking. The CAB reviewed and approved the revised instructions. The revision read as follows. The wording has small alterations, but the meaning is unchanged: “***Purpose:*** *The tool's purpose is to identify what the most important considerations are for you in deciding on treatment for metastatic breast cancer.* ***Instructions:*** *The next page has categories that women with metastatic breast cancer identified as important. Write in the categories from most important (#1) to least important (#9). If you have things that are important to share with your clinician not in the tool, write them in the box at the bottom*.”

### Usability and Format of the VAsT

Regarding the VAsT format, participants were asked to comment on the tool’s design and format (e.g., paper vs. electronic). Most participants preferred electronic delivery, while many also indicated that there should be an option to use a paper version to improve accessibility and equity for others. One care partner described the important rationale behind offering a paper copy:

*“We’re east of [Highway], and that’s a digital wasteland. There’s so many people out here that don’t have access to computers. [Name] has actually opened our home to people [they’ve] met at the local cancer center that couldn’t apply for assistance or financial aid or whatever because they didn’t have a computer, and they didn’t know how to work a computer….For that reason, I would say paper.”* [Care partner, ID73]

Other considerations identified across interviews included ensuring it remained accessible on mobile devices and the user experience of ranking domains.

Nearly all participants stated that the VAsT should be administered at the beginning of treatment. Opinions varied on how frequently the VAsT should be completed, but completing it multiple times over the course of treatment was the nearly exclusive view. Most felt it would be appropriate to administer it before important treatment decisions, while others felt it could be used before every appointment. This reflected the recognition that women’s values can change over the course of treatment. One clinician detailed points of care where the VAsT would be useful over time:

*“Then certainly, as things go along, especially maybe at the time, the big decision time points at scan reviews or times where we have to switch to a different line of therapy, having a reassessment or a reevaluation if this has changed.”* [Clinician]

### Communication and Clinical Utility of the VAsT

Communication and clinical utility were the most robust themes identified. Participants indicated that the VAsT would be most effective at prompting women with mBC to reflect on their values and facilitating communication between women and care teams. Some participants felt the tool would also help communicate to their care partners what they wanted to prioritize in treatment, while others felt they had already communicated this effectively and that the VAsT would be more applicable to others. For example, one woman with mBC highlighted the value of the VAsT for communication with clinicians: *“…this would be good for someone who just is not there with advocating for themselves. This is something that they could use ’cause these are good questions.”* [Woman with mBC, ID14] Clinicians indicated that having the VAsT completed prior to appointments where treatment decisions were likely to be made would help them discuss the risks and benefits of specific treatment options more effectively.

Additionally, participants identified two notable factors that may affect communication with their clinicians about values entirely: (1) perceived quality of care, and (2) trust in clinicians. Some patients described poor experiences with their treatment in the past and perceived poor communication with their care team that led to a lack of trust. One woman with mBC recalls:

*“One of 'em even asked me did I come to the appointment that day to listen? Yeah. I said, “What in the world?” and then I tried to change her and then I was told I couldn't. That was when I left and went out of state. I said this is getting crazy at this point. My experience was, I would hope that most people don't have it, but I'm afraid more people do than we realize. It can get very ugly in those rooms, it really can and that's that.”* [Woman with mBC, ID15]

This lack of trust also shaped their perception of the quality of care. Participants identified the VAsT as having the potential to help build trust between women with mBC and their care team; this can be achieved by facilitating discussions about treatment decisions that better incorporate values and lead to perceptions of improved quality of care. A hypothetical example was given:

*“Well, I think it—if they know these things [on the VAsT], it almost helps you get to know the person a little better, quicker. They might have said like, “Side effect—minimize and manage side effects of treatment,” that would—that's when the palliative care nurse comes in. If that's number one, the doctor might say, “Okay, I know how to help you with this. I'm gonna refer you to this nurse, that's going to help you with your side effects and manage your treatment—and manage your pain.””* [Woman with mBC, ID16]

## Discussion

This qualitative study, conducted with women with mBC, care partners, and clinicians, confirmed the relevance of VAsT domains, identified areas for further refinement, and described perceptions of VAsT utility. Key findings included: 1) recommended use of the VAsT at the beginning of treatment and periodically, considering treatment decision-points and changing values; 2) the utility of VAsT to facilitate reflection of values and communication regarding treatment decisions for women with mBC; and 3) the potential of VAsT to build trust and improve quality of care between women with mBC and their clinicians. The themes identified in these cognitive interviews informed the refinement of the VAsT and indicated the potential utility of incorporating patients’ values into their treatment decisions.

While most participants indicated that the VAsT instructions and domain clarity were well delivered, some feedback indicated that the placement of the instructions and the purpose of the VAsT could be improved. The research team incorporated the feedback from these interviews and decided to place the purpose of the VAsT and instructions on a separate page to be read before engaging with the VAsT itself. These improvements will aid understanding of how to use the tool as its practical implementation is studied in the future.

Further, participants indicated that VAsT’s utility would be optimized by having it available in an electronic format that can be incorporated into medical records, which allows for their care team to review responses from women with mBC prior to appointments. This may complement other information in the electronic medical records, leading to improved quality of care.^23^ However, as reflected in these interviews, it is important for care teams to recognize that having a paper format available would increase accessibility, given differing preferences for delivery format and the need for paper options to be readily available in lower-resource communities. This aligns with prior research on healthcare access in rural communities, which may face structural barriers to care, including a lack of broadband internet.^24^ Participants also noted that a patient’s values in their care may change over time, depending on disease stability or personal circumstances. Therefore, the prevailing opinion was to administer the VAsT at the beginning of treatment and, at a minimum, prior to appointments where potential changes in treatment decisions may be discussed. Considering the potential for changes in symptoms and the person’s health while living with metastatic disease,^5^ repeated use of the VAsT throughout the care trajectory was recommended. Therefore, future research on the VAsT should investigate its feasibility and efficacy when implemented in clinical care at multiple time points.

The themes identified in this study indicate that participants agreed that VAsT achieves its aims of prompting patients to reflect on their values regarding their course of treatment and facilitating communication of these values with their care partners and care team. This has the potential to address an unmet need for treatment decision conversations that may occur simultaneously in the clinical setting.^25^ Additionally, this aligns with the standards and practice recommendations from the Multinational Association for Supportive Care in Cancer (MASCC) and American Society of Clinical Oncology (ASCO), which emphasize that women with mBC should be active collaborators with clinicians to facilitate value-congruent care.^26^ Understanding whether a patient has financial concerns that may affect their treatment, not wanting to appear sick, or prioritizing maximizing time away from appointments are all clinically relevant values to incorporate into care for women with mBC. Based on these findings, the potential of VAsT to facilitate clear communication of women with mBC’s values is likely to improve trust between women with mBC and their care team and enhance their perceptions of the quality of care they receive. As one participant stated, the VAsT can “*provide insight into who you are as a human being*”. Further, given the multiple therapeutic options for patients with mBC, often with comparable efficacy, patient values and preferences are key considerations in treatment selection, and the VAsT helps elucidate these values and preferences for shared decision-making.

Strengths of this study include robust data collected from three groups of participants with perspectives on the utility of the VAsT: 1) women with mBC, 2) care partners, and 3) clinicians. Additionally, 43% of women with mBC were Black or African American, and participants were enrolled from multiple locations in the United States. Limitations include the care partners being all male, which may not be representative of all care partners for women with mBC, and the lack of male patient participants, although these are a minority of patients with mBC. Furthermore, all clinicians were enrolled at the same cancer center, which may influence their perspectives on the tool’s clinical utility within existing workflows and limit generalizability to other clinical contexts. Finally, although the changes to the VAsT were not substantive, the instructions and format were modified without a follow-up round of interviews to confirm that these changes improved understanding of the VAsT.

The findings from this study confirm the VAsT domains and have aided in further refinement of the tool. The VAsT has the potential to improve the shared decision-making process of treatment decisions for women with mBC. Future research on the VAsT should include investigating the feasibility of implementing the VAsT in care for women with mBC and assessing the tool’s efficacy on decision-making, preference-concordant care, and communication longitudinally.

## Conclusion

In summary, this study aimed to understand the perspectives of women with mBC, care partners, and clinicians when using the VAsT to better understand the perceived utility and usability, confirm the domains of the VAsT, and identify further areas of refinement of the VAsT. This was achieved through cognitive interviews with 14 individuals. Findings from this study have been incorporated into relevant improvements to the VAsT and confirm the potential for improved shared decision-making in the treatment decisions of women with mBC. Future longitudinal research would help assess the feasibility of implementing the VAsT and its efficacy in facilitating the incorporation of values into treatment decisions.

## Supplemental Material

Supplemental material - “Insight Into who you are as a Human Being”: Perceptions of the Utility and Usability of a Values Assessment Tool (VAsT) for Women With Metastatic Breast Cancer Supplemental material for “Insight Into who you are as a Human Being”: Perceptions of the Utility and Usability of a Values Assessment Tool (VAsT) for Women With Metastatic Breast Cancer by Robert Crowder, Victoria Crowder, Soroush Fariman, Natasha Joglekar, Danielle Anderson, Juanita Miller, Stephanie Walker, Emily M. Ray, Shaunta Ford-Pierce, Bryce B. Reeve, Antonia Bennett, Melissa Mazor, Allison M. Deal, Lorinda A. Coombs in Cancer Control

## Data Availability

Data is not publicly available due to the potential identification of the participants through interview topics.*

## Appendix

### Abbreviations

CAB: Community Advisory Board
IRB: Institutional Review Board
mBC: Metastatic breast cancer
NPT: Normalization Process Theory
VAsT: Values Assessment Tool
