# Supplementary material for: “Insight into who you are as a human being”: Perceptions of the Utility and Usability of a Values Assessment Tool (VAsT) for Women With Metastatic Breast Cancer
Source: Cancer Control. 2026 Jul 28;33:10732748261466394. doi: 10.1177/10732748261466394 (PMC13420118; doi:10.1177/10732748261466394)
Supplement: Supplemental material - “Insight Into who you are as a Human Being”: Perceptions of the Utility and Usability of a Values Assessment Tool (VAsT) for Women With Metastatic Breast Cancer [file sj-pdf-1-ccx-10.1177_10732748261466394.pdf]

“Insight into who you are as a human being”: Perceptions of the Utility and Usability of a Values Assessment Tool (VAsT) for Women with Metastatic Breast Cancer

Supplementary File

Interview Guides

Interviews with Women with mBC

Part 1: Before viewing the VAsT, ask a general question about what values/factors the participants believe are essential to deciding on their cancer treatment.

Part 2: View VAsT and “think out loud” when reading and ordering values.

- Probe on the meaning of each item/category.
- Discuss their approach to ordering each item/category.
- Are directions clear?
- How user-friendly do you think the tool should be, and what would make it user-friendly?

Part 3: Post completing the VAsT.

1. What is your overall impression of the tool?
2. Discuss what changes you might make to the tool.
    a. Is there any area that would be particularly helpful, or conversely, not an accurate or helpful topic listed?
    b. What would it be if you could change one thing about the concept or design?
3. Discuss the strengths and limitations of the tool for use in cancer care.
    a. How might you use this tool in your clinic visit with your care team?
    b. How could this tool help you or someone else navigate the cancer journey?
    c. How might this tool improve communication with healthcare providers or care partners?
    d. Are you aware of similar tools or resources? How could this one differ or improve upon those?
    e. How likely would you be to use this tool if it were available?
    f. What factors might influence your decision to use or not use this tool?
    g. How would you like to use this tool (e.g., as an app on the phone, on the computer, or written)?
    h. When would you want to use this tool, at the beginning of the diagnosis and/or when treatment changes are necessary?

Interviews with Care Partners of Women with mBC

Part 1: Before viewing the VAsT, ask a general question about what values/factors the care partner believes are essential in treatment decisions and communication.

For example, I want to learn a little bit about you. Can you tell me about yourself? What role do you play in supporting [the patient] and their cancer care?

Part 2: Review VAsT and “think out loud” when reading and ordering values.

- Probe on the meaning of each item/category.
- Discuss their approach to ordering each item/category.
- Are directions clear?
- How user-friendly do you think the tool should be, and what would make it more user-friendly?

Part 3: Post completing the VAsT.

1. What is your overall impression of the tool?
2. Discuss what changes you might make to the tool.
    a. Is there any area that would be particularly helpful, or conversely, not an accurate or helpful topic listed?
    b. What would it be if you could change one thing about the concept or design?
3. Discuss the strengths and limitations of the tool for use in cancer care.
    a. How might you support [Patient's name] in using this tool in their clinic visit with the care team?
    b. How could this tool help [Patient's name] or someone else navigate their cancer treatments?
    c. How might this tool improve communication with healthcare providers or between yourself and [Patient's name]?
    d. Are you aware of similar tools or resources? (if they say yes, ask about details such as the names of the other tools) How could this one differ or improve upon those?
    e. How likely do you think [Patient's name] would use this tool if it were available?
    f. What factors do you think might influence [Patient's name] decision to use or not use this tool?
    g. How would you prefer to access this tool (e.g., as an app on the phone, on the computer, or written)?
    h. When do you think it would be most helpful to use this tool, at the beginning of the diagnosis and/or when treatment changes are necessary?

Interviews with Oncology Clinicians

We would like to hear from you as an oncology clinician about whether the information contained within a completed values assessment tool would help you understand what is important to patients with metastatic breast cancer.

After showing them the VAsT, we will ask the following questions:

1. Would this information be important to you to provide patient centered care for women with metastatic breast cancer?

2. Are there any domains within the tool that are missing (i.e. that you would like to know in order to tailor your patient care?)

3. When in the patient's care trajectory would you like to receive this information about a patient with mBC? (e.g., before your first appointment with a new patient, later in the treatment

sequence?) And would you find it helpful to review/discuss more than once, over time as you care for a patient?

4. How would you like to know this information? Receive an electronic copy of the completed tool before the visit, a printed copy before the visit, or have the patient and/or caregiver bring it to clinic and discuss their results with you etc.

5. What would be the most efficient and effective way to integrate a values assessment tool into clinic flow?

    a. Patient feedback has suggested that having access to previously completed tools would be helpful for them to understand the evolution of their values—would that be useful to you?
